# 텍스트 네트워크 분석을 이용한 조산 경험 이야기의 시각화

**DOI:** 10.4069/kjwhn.2020.08.08

**Published:** 2020-08-28

**Authors:** Jeung-Im Kim

**Affiliations:** School of Nursing, Soonchunhyang University College of Medicine, Cheonan, Korea; 순천향대학교 의과대학 간호학과

**Keywords:** Clinical presentation, data visualization, Personal narrative, Premature obstetric labor, 임상 표현, 자료 시각화, 개인 경험 이야기, 조기 진통

## Abstract

**Purpose:**

This study aimed to identify the components of preterm birth (PTB) through women’s personal narratives and to visualize clinical symptom expressions (CSEs).

**Methods:**

The participants were 11 women who gave birth before 37 weeks of gestational age. Personal narratives were collected by interactive unstructured storytelling via individual interviews, from August 8 to December 4, 2019 after receiving approval of the Institutional Review Board. The textual data were converted to PDF and analyzed using the MAXQDA program (VERBI Software).

**Results:**

The participants’ mean age was 34.6 (±2.98) years, and five participants had a spontaneous vaginal birth. The following nine components of PTB were identified: obstetric condition, emotional condition, physical condition, medical condition, hospital environment, life-related stress, pregnancy-related stress, spousal support, and informational support. The top three codes were preterm labor, personal characteristics, and premature rupture of membrane, and the codes found for more than half of the participants were short cervix, fear of PTB, concern about fetal well-being, sleep difficulty, insufficient spousal and informational support, and physical difficulties. The top six CSEs were stress, hydramnios, false labor, concern about fetal wellbeing, true labor pain, and uterine contraction. “Stress” was ranked first in terms of frequency and “uterine contraction” had individual attributes.

**Conclusion:**

The text network analysis of narratives from women who gave birth preterm yielded nine PTB components and six CSEs. These nine components should be included for developing a reliable and valid scale for PTB risk and stress. The CSEs can be applied for assessing preterm labor, as well as considered as strategies for students in women's health nursing practicum.

## Introduction

### 연구 필요성

조산은 임신 37주전에 일어난 출산으로[1], 조산아는 발달장애 및 학습능력장애, 행동장애 등의 동반으로[2,3] 임신 여성과 가족의 삶의 질 뿐만이 아니라 사회·경제적으로 부정적인 영향을 미치며[2] 임신 중 불안은 임신기간 단축으로 태아의 신경계 발달에 좋지 않은 영향을 주기 때문에[3] 고위험 임산부들이 갖는 조산 가능성에 대한 두려움이 크다[2]. 조산의 위험요인은 비정상적 장 기능, 28주 이전 조기 진통을 진단받은 시기, 사회적 지지 부족[4], 조기 진통, 조기 양막파열, 건강하지 못한 생활습관, 의학적 위험요인, 생식기계 위험요인 등으로 다양하고[5], 높은 산전 스트레스나 높은 불안으로 위험도가 높아지며[4], 예상하지 못한 상태에서 급박하게 진행되므로 근거 중심 예방간호가 필요하다[6].

조산의 전구인자에 따른 상태는 (1) 진통이 시작되어 유도분만을 하거나 진통이 일어나기 전 제왕절개를 해야 하는 산부나 태아의 상태, (2) 양막이 온전한 상태에서 자연적으로 발생한 조기 진통, (3) 만삭 전 양막파열과 함께 오는 조기 진통의 세가지 유형이 있음이 보고되었다[7]. 임신 37주 이후 임산부는 하강감(lightening), 가진통(false labor), 이슬(show), 양막파열(rupture of membrane), 자궁경부 숙화(ripening)와 같은 분만 전구 증상을 경험하는데, 37주 이전의 조산에서는 자궁경부 변화와 함께 자궁 수축이 있으며 골반 압박감(pelvic pressure), 월경통 같은 조임(menstrual-like cramps), 물 같이 흐르는 질 분비물(watery vaginal discharge), 그리고 하부요통(low back pain)같은 증상이 나타날 수 있다. 그러나 이는 정상 임신에서도 흔하고 모든 참여자에 나타나는 것이 아니기 때문에[8,9] 객관적으로 평가하기 어려운 속성이 있다. 그러므로 조산 가능성이 있는 임부는 신체적, 심리적 요인을 포함하여 면밀하게 평가할 필요가 있다[4,9-11].

특히 조산 관련 요인들이 모든 임산부에게 균등하게 영향을 미치는 것은 아니며 통증을 동반하거나 동반하지 않은 자궁 수축이 정상 분만에서도 나타날 수 있어 임산부나 의료진들은 조산의 전구 증상인 자궁 수축의 증상들을 간과하기 쉽다[9]. 따라서 간호사는 고위험 임산부가 통증을 호소할 경우 자궁 수축 여부와 관계없이 조산이 임박할 때 나타나는 증상인 점[9,12]을 알고 적절하게 대처해야 한다.

이와 같이 조산이 일어나기 전 진통과 양막파열이 반드시 동반되는 것은 아니기 때문에 조산을 예방하기 위해서는 임산부의 임상증상 표현(clinical symptom expression, CSE)을 이해하는 것이 중요하다. 그러나 여성건강간호학 교과서[8]와 산과학[9]에 나타난 조산 관련 공통 증상은 ‘월경 증상과 유사한 경련, 골반 압박감, 하부요통 및 질 분비물’로 기술되어 있고 임산부의 임상증상 표현으로 이해하기 어려웠다.

간호사가 환자의 임상증상 표현을 이해하는 것은 간호 행위를 결정하는 중요한 요소이다. 특히 시각을 다투는 조기 진통이나 조기 양막파열의 임상증상 표현을 이해하는 것은 조산 위험 임부의 정확한 간호 사정에 필요하므로 조산 전 임산부들의 이야기에서 임상증상 표현을 추출하는 연구를 할 필요가 있다.

임산부의 행동과 정서를 표현하는 비구조화된 텍스트 속에 포함된 임상증상 표현들이 신체적 심리적 요인들과 어떤 관계 속에 있는지 분류하기는 쉽지 않다. 텍스트 네트워크 분석방법은 네트워크 이론을 기반으로 코딩(coding), 시각화(visualization), 군집화(clustering) 등의 기술을 적용하여 참여자의 이야기를 시각화할 수 있다[13]. 키워드 텍스트 네트워크 분석이나 워드 클라우드(word cloud)를 활용한다면 객관적인 분석이 가능해지고 연구의 주제와 동향을 다각도로 조명해 볼 수 있다[14]. 그러므로 본 연구에서 조산 산모의 개인적 경험 이야기에서의 구성요인(components) 도출은 키워드 네트워크 분석을 적용하는 것이 적합하다.

이야기(narrative)란 시간적으로 순서가 있고, 어떤 의미를 전달하는 경험이나 사건에 대한 설명이며[15], 개인적 경험 이야기는 이야기 화자 혹은 이야기 화자가 알고 있는 누군가가 경험한 실제의 특정한 경험에 대한 설명이므로[16] 조산 산모가 경험한 것을 연구자에게 제공하는 것은 개인적 경험 이야기에 해당한다.

이에 본 연구에서는 조산한 산모를 대상으로 대화형 스토리텔링(interactive storytelling)을 통해 수집된 비구조화된 자료를 키워드 네트워크 분석방법의 하나인 시각화 분석(bibliometric mapping)으로 조산 위험의 구성요인과 임상증상 표현을 추출하고자 하며 추출된 구성요인은 조산 위험을 사정하는 도구 개발의 기초자료로 사용할 예정이다. 이 연구를 통해 얻어진 임상증상 표현은 산과 외래 및 고위험 임산부 집중치료실(maternal fetal intensive care unit, MFICU)/분만실 간호사 뿐만 아니라 여성건강간호학 실습 중 산과 실습을 하는 학생들이 만삭 전 출산하는 여성의 임상증상을 이해하고 필요한 간호를 계획하는 데 도움이 될 것으로 기대한다.

### 연구 목적

본 연구는 임신 28주에서 37주 전에 출산한 조산 산모를 대상으로 조산이 있기까지의 경험을 비구조적 자료로 수집하여 텍스트로 만들어 구성요인을 추출하고, 키워드 네트워크 분석(키워드 분석과 시각화 분석)을 통해 임상증상 표현을 시각화하는 것을 목적으로 하며, 구체적인 목표는 다음과 같다.

1) 참여자의 조산 전 경험 이야기에 나타난 의미 있는 구성요인을 파악한다.

2) 참여자의 조산 전 경험 이야기에서 추출된 임상증상 표현을 시각화한다.

## Methods

Ethics statement: This study was approved by the Institutional Review Board of Soonchunhyang University College of Medicine (10408 75-201905-SB-026). Informed consent was obtained from the participants.

### 연구 설계

본 연구는 임신 28주 이상 37주 전에 신생아를 출산한 조산 산모를 대상으로 대화형 스토리텔링으로 면담한 비구조화된 개인적 경험 이야기를 키워드 네트워크 분석인 시각화 분석을 하여 조산 관련 임상증상 표현을 추출하기 위한 내용 분석 연구이다. 주된 질문은 ‘조산에 영향을 준 것은 무엇이었는가?’와 ‘조산이 일어나기 전 경험한 증상은 무엇인가?’였다.

### 연구 대상

본 연구의 참여자는 임신 28주 이상 37주 전에 MFICU에 입실하여 신생아를 출산한 조산 산모이고, 제외 기준은 임신 37주 0일 이후에 신생아를 출산한 산모이다. 참여자 선정은 순천향대학서울병원 산과 교수가 참여자에게 연구 목적을 설명한 후 참여 희망 산모의 연락처를 받았다. 연구자는 전화로 연구 목적을 설명한 후 참여 의사가 있는 조산 산모와 면담 일정을 정하였다. 면담 전 설명문을 읽은 후 동의할 경우 서명을 하고 면담에 참여하였다. 본 연구 참여자는 조산을 한 산모이기 때문에 특정 성별(여성)만을 대상으로 하였다.

### 자료 수집

자료 수집은 2019년 8월 16일부터 12월 4일까지 이뤄졌다. 면담 일정을 정한 후 산모가 원하는 시간과 장소에서 면담을 하였다. 아기를 돌봐야 하는 산모들이어서 집에서 면담을 하였고, 1시간 정도 나올 수 있는 경우 카페에서 만났다. 본 연구와 관련하여 개인적인 조산 경험에 대한 이야기(personal narratives)를 나누어 간호사가 조산 임박 증상을 이해할 수 있도록 참여자들의 임상증상 표현을 연구하고자 한다는 점을 설명하고, 정답이 없고 개인의 경험을 이야기해 주도록 부탁하였다. 면담 시작 시 녹음하였고 면담이 종료되면 바로 당일에 문서로 작성하였다.

### 자료 분석

텍스트 네트워크 분석은 텍스트 분석 프로그램 MAXQDA 2020 (ver. 20.0.7; VERBI Software, Berlin, Germany)을 이용하였다. MAXQDA 2020은 한 문장에 관련된 코딩을 입력하고 수정하는 것이 가능한 프로그램으로, 수집한 자료에서 키워드를 추출하고 분석 및 시각화하는 과정으로 이뤄졌다. 수집한 text 문서를 pdf 문서로 변환한 면담 자료를 읽으면서 의미 있는 단어나 문장에 코딩을 하여 전체 코드 시스템을 완성하였다. 내용 분석은 분석자료에 코딩된 단어의 출현 빈도(term frequency)를 계산하는 과정을 거쳐 중심 키워드를 시각화하였다. 구체적인 내용 분석 단계는 다음과 같다.

먼저 조산 산모와의 비구조화된 면담 자료를 데이터베이스화 하였다. PDF로 변환한 자료를 읽고 코딩하면서 의미 있는 이야기에는 메모를 하고 새로운 코딩을 하면서 상위 코드로 넣거나 새로운 코드를 만들었다. 이러한 과정의 반복을 통해 코드 체계(code system)를 완성하여 구성요인을 도출하였다. 다음으로 coded text segments를 code matrix browser로 코드 빈도를 계산하여 중심 주제어를 추출하였다. 그리고 참여자의 조산 경험 이야기의 텍스트를 이용하여 similarity matrix 분석을 하였다. 마지막으로 Microsoft Excel (Microsoft, Redmond, WA, USA)의 spread sheet에 추출된 주제를 분석자료로 이용하여 핵심 키워드의 출현 빈도와 워드 클라우드로 임상증상 표현을 시각화하였다.

## Results

### 참여자의 특성

본 연구 참여자는 11명으로 모두 37주 0일 이전에 조기 출산한 산모이다. 참여자의 평균 연령은 34.6 (±2.98)세이고, 염산 리토드린 주사(ritodrine HCl injection; Yutopar, JW Pharmaceutical, Seoul, Korea)는 8명 참여자의(72.7%), 아토시반 주사(atosiban injection; Tractocile, Ferring, Seoul, Korea)는 5명(45.5%)이었고, 조기 양막파열은 8명(72.7%)에서 있었다. 자연 분만 5명(45.5%), 제왕절개 분만이 6명(54.5%)이었다. 출산 후 신생아의 즉각적인 생존 여부를 평가하는 1분 Apgar 점수와 장기간의 생존 및 신생아의 신경학적인 상태를 예측하는 평가인 5분 Apgar 점수[8] 결과를 보면 1분 Apgar 점수는 0–3점 1명, 4–6점 1명, 7–10점 9명으로 평균 7.91 (2.21)점이었으며 5분 Apgar 점수는 11명 신생아 모두 8점 이상으로 평균 9.55 (.82)점이었다(Table 1).

### 조산 전 경험의 구성요인

**Figure 1.**
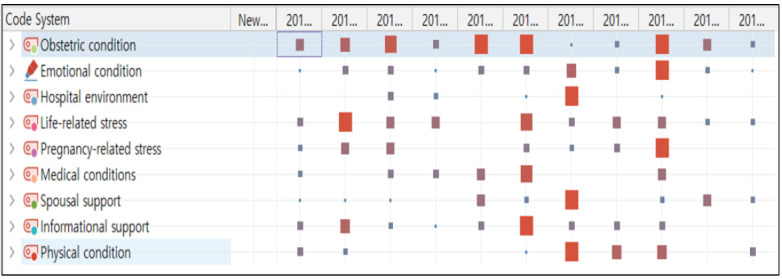
Components of segmented code-by-code matrix.

임신 28주 이상부터 37주 0일 이전에 조산한 산모들이 조산 전 경험한 증상과 상황에 대하여 대화형 스토리텔링으로 얻어진 비구조화된 자료에 대해 코드 체계로 분석하여 조산 전 경험의 구성요인을 파악한 결과 총 364개의 code로 분류되었으며, 수차례의 코딩을 한 결과 산과적 상태(126개), 정서적 상태(66개), 병원 환경(14개), 생활 스트레스(24개), 임신 스트레스(19개), 내과적 문제(16개), 남편의 지지(27개), 정보의 지지(40개), 신체적 상태(32개)의 9개의 구성요인으로 분류되었다. 이중 상위 3개는 산과적 상태, 정서적 상태, 정보 지지로 나타났다(Figure 1).

**Figure 2.**
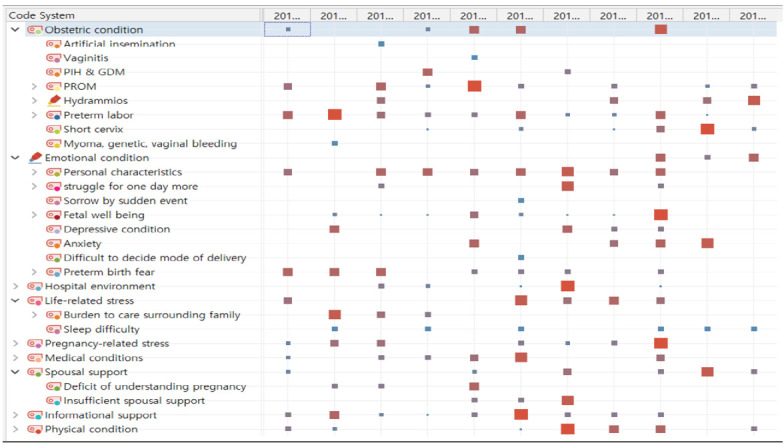
Frequency of segmented code-by-code matrix.

다음으로 coded text segments를 모두 열어 code matrix browser로 코드 빈도를 계산하여 symbol size로 시각화한 결과, 66개의 코드 중 노드(node)가 컸던 코드는 조기 진통 증상, 개인 성격, 조기 양막파열로 나타났다. 노드 크기가 상위 3개보다 작았지만 참여자의 절반 이상이 경험한 코드는 ‘짧은 경관 길이’, ‘조산의 두려움’, ‘태아 안녕’, ‘수면의 어려움’, ‘불충분한 배우자의 지지’, ‘불충분한 정보 제공 지지’ 그리고 ‘신체적 힘듦’으로 나타났다(Figure 2). 조산으로 인한 태아 건강에 대한 걱정으로 참여자들의 삶은 ‘하루하루 버티기’로 추출되었다. 참여자들의 경험의 유사성을 similarity matrix로 검토한 결과 .54에서 .78로 높은 유사성을 보였다.

### 조산 전 경험에서 추출된 임상증상 표현

**Figure 3.**
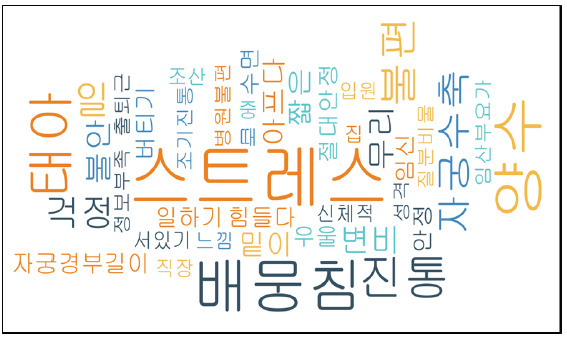
Word cloud of clinical symptoms expressions for preterm birth mothers; 40 words expressed at least three times (total, 323 words).

조산 산모의 조산 전 경험에서 추출된 임상증상 표현은 Microsoft Excel의 spread sheet에 정리된 284개의 주제 가운데 ‘했다’, ‘한다’, ‘잘’, 있다’, ‘들었다’와 같은 반복어를 제외하고 비슷한 주제를 통합하는 과정을 거쳐 270개 주제로 분류하였다. 이 주제들에 들어있는 총 530개 단어를 대상으로 분석 기준을 ‘2회 이상 언급’과 ‘75개 단어’로 제한한 워드 클라우드 분석과 ‘3회 이상 언급’과 ‘40개 단어’를 분석 기준으로 한 결과, 총 323단어(type–token ratio=0.0755)로 나타났으며, 이 가운데 스트레스가 가장 많았고 배 뭉침(가진통), 양수 터짐, 태아 걱정, 진통(진진통), 자궁 수축이 두드러지게 나타났다(Figure 3).

다음 단계로 270개의 주제를 키워드 출현 빈도로 분석한 결과 상위 6개는 스트레스(15회), 배 뭉침(가진통) (13회), 양수 터짐(13회), 태아 걱정(11회), 진통(진진통) (10회), 자궁 수축(8회)으로 나타났다. 상위 6개는 모두 정서적 상황과 산과적 상황의 코드 체계에 포함되는 것으로 이를 뒷받침하는 이야기는 Supplementary data와 같다.

마지막으로 임상증상 표현 분석 결과 중 조기 진통 및 자궁 수축의 임상증상을 살펴보면 ‘허리가 아프다’, ‘밑이 빠질 것 같다’. ‘축구공처럼 배가 빵빵하다’. ‘톱으로 써는 것 같다’, ‘전봇대로 잡아빼듯 깊숙이 아팠다’ 등으로 나타났다. ‘질 쪽에 조임 같은 느낌’, ’조이고 딱딱해지기만 했어요’, ‘항문을 찌르는 느낌, 항문으로 내려가는 느낌이 있었어요’ 등 조기 진통 증상 표현이 개인마다 달랐다. 또한 자궁 수축의 표현은 ‘미친듯이 아팠어요’, ’좀더 배꼽의 왼쪽 거기가 태반 쪽인가 움직이지 못하고 걷지도 못할 정도로 아팠어요’, ‘너무 아파 주저앉았어요’, ‘아침에 괜찮다가 밤에 새벽에 특히 수축이 많이 왔어요’, ‘와이존이 빠질 것 같은 느낌’, ‘배가 사르르 아픈 느낌’, ‘엉덩이 쪽이 아팠어요’와 같이 나타났다. 양수인지 파악하는 방법으로 ‘락스 냄새가 났다’는 점을 통해 양수의 냄새에 대한 특성을 알 수 있었다(Supplementary data).

## Discussion

본 연구는 조산한 산모가 경험한 개인적 이야기를 토대로 조산과 관련된 임상증상 표현을 찾기 위해 내용 분석을 실시하고 키워드 네트워크를 파악하여 임상증상 표현을 시각화하였다.

본 연구 결과 조산 경험의 구성요인은 산과적 상태, 정서적 상태, 병원 환경, 생활 스트레스, 임신 스트레스, 내과적 문제, 남편의 지지, 정보의 지지, 신체적 상태의 9개로 추출되었다. 이는 건강하지 못한 생활습관, 의학적 위험요인, 생식기계 위험요인 등의 분류[6], 임신력(과거 조산 경험), 임신특성(다태임신, 질 출혈, 심리·사회적 스트레스, 임신 관련 우울, 감염), 생물학적 유전적 지표(biological and genetic markers; 양수, 소변, 경관 점액, 질 분비물 같은 생물학적 액체, fetal fibronectin 등)의 분류[7]와 세부적인 차이를 보였다. 여성건강간호 교과연구회[8]에서도 인구학적 요소, 산과적 과거력, 현재 임신 관련 산과적 요소, 내과적 과거력, 생활습관 및 환경적 요인 등으로 보고한 결과와 비교해볼 때 본 연구 결과의 구성요인 중 ‘정서적 상태, 병원 환경, 생활 스트레스, 남편의 지지, 정보의 지지’가 포함되어 있지 않았음을 확인할 수 있었다. 이는 Ahn 등[11]과 Raju [12]의 연구와도 유사한 것으로, 조산 전 경험의 구성요인이 구체적일 때 조산 위험 임산부에게 체계적인 간호를 제공할 수 있으므로 체계적인 문헌 고찰을 통해 구성요인을 표준화할 필요가 있다. 또한 본 연구 참여자의 평균 연령은 34.6 (±2.98)세로 2019년 우리나라 조산 산모의 평균 연령인 33.5세[17]보다 많았으며 조산 위험요인 중 인구학적 연령 요소인 20세 이하 40세 이상[8]의 기준에는 속하지 않았다는 점에서 인구학적 요소 중 연령에 대한 연구가 필요하다.

다음으로 본 연구에서 조산 산모의 이야기에서 키워드 네트워크 분석 결과, 스트레스가 가장 많았고 이어서 배 뭉침(가진통), 양수 터짐, 태아 걱정, 진진통, 자궁 수축 순으로 나타났다. 교과서[8]에서 제시한 전구 증상과 비교할 때 ‘통증’과 ‘자궁 수축’이라는 공통 키워드만 유사한 것을 볼 수 있다. 본 연구에서 스트레스가 가장 다빈도로 나타난 점과 조기 양막파열 전 생활 스트레스나 신체적 스트레스를 경험한 점은 조산 산모의 20%가 임신 중 스트레스에 노출되었고, 임신 3기의 스트레스와 임신 관련 불안이 조산과 관련된다는 연구들[18-20]과 일치하는 결과이다. 특히 누적된 사회·심리적 스트레스 변인은 조산 위험요인과 함께 평가되어야 한다[20]. 그러나 지금까지 국내에서 이뤄진 조산 관련 간호학 연구는 조기진통 임부의 조산 발생 영향요인, 스트레스와 상관관계[4,21], 입원 중인 임산부의 스트레스 대처 유형[22], 조산의 현상학적 연구[23] 등으로, 스트레스의 속성을 다룬 연구가 많지 않아 조산의 예방과 관리에 있어 임상증상 표현과 연결된 조산 위험 스트레스 척도의 개발이 선행될 필요가 있다.

본 연구 결과 조기 진통과 자궁 수축이라는 임상증상 표현은 개인적 차이를 반영한 구체적인 표현인 ‘톱으로 써는 것 같다’, ‘전봇대로 잡아빼듯 깊숙이 아프다’, ‘와이존이 빠질 것 같다’ 등으로(Supplementary data), 이는 여성건강간호학 교과서에 나타난 ‘월경통과 유사한 복통이나 장의 통증, 골반의 압력, 설사, 하부요통 및 질 분비물 증가’[8]나 산과학에서 제시한 ‘규칙적이거나 빈번한 복부 조임(수축)’, ‘지속적이고 묵직한 허리 통증’, ‘골반이나 하복부가 눌리는 느낌’, ‘가볍게 복부가 조여짐’[11,24,25]과 비교했을 때 조산 전 가진통과 자궁 수축의 증상에 대한 참여자의 임상증상 표현은 다양함을 확인하였다. 이러한 다양한 임상 표현은 임박한 조산을 예측할 수 있는 조산 위험 도구 개발에 활용하여야 할 것이다.

의학 실습교육에서는 75개 임상 표현(clinical presentation)으로 시작하였다가[26,27] 120개(제2판)로 증가시켰다[28]. 이 가운데 산부인과 임상 표현은 무월경(amenorrhea), 불임/난임(infertility), 산전 관리(antepartum care), 외음부덩이(pudendal mass), 요실금(urinary incontinence), 월경통/골반통(dysmenorrhea/pelvic pain), 젖흐름증/유두 분비(galactorrhea/nipple discharge), 질 분비물(vaginal discharge), 질 출혈(vaginal bleeding), 폐경(menopause), 피임(contraception), 허리 통증(low back pain)의 12개이다[28]. 이들 산부인과 임상 표현과 본 연구 결과를 비교하였을 때 ‘스트레스’, ‘배 뭉침(가진통), ‘태아 걱정’, ’양수 터짐’, ‘진진통’, ‘자궁수축’ 등은 조산 전 임산부의 상태를 사정할 수 있는 구체적인 임상증상 표현이라고 볼 수 있다. 본 연구 결과 조산 전 경험에서 나타난 임상증상 표현은 여성건강 교과서에서 제시한 것[8]이나 산과학[9]에서 제시한 골반 압박감, 월경 증상과 유사한 경련, 물 같이 흐르는 질 분비물, 허리 통증과 같은 불편감과는 차이를 보여 이들 표현에 대한 추가적인 연구가 필요하다. 그럼에도 불구하고 조산 위험으로 가정 내 관리를 하는 임산부의 경우 자궁 수축과 배 뭉침을 구분하기 어려워한다는 점[29]을 고려할 때 스토리텔링 기반 임상증상 표현의 코딩화는 조기 진통 유무를 구분하는 데 기여할 것으로 예상된다.

본 연구에서는 MAXQDA 프로그램을 활용한 키워드 네트워크 분석방법을 적용하였다. 이 연구방법은 회복력의 연구동향[13], 학술지 분석[14], 조기 진통과 출산의 결정요인[30] 등과 같이 키워드 분석에 이용되었다. 키워드 네트워크 분석에서 구성요인의 분류는 질적 연구에서 주제 도출과정과 같이 연구자의 수준에 달려있다고 볼 수 있다. MAXQDA 프로그램을 이용한 분석은 객관적으로 분류될 때까지 전체 텍스트를 코드 체계 안에서 분류(sorting)와 코딩을 수차례 반복하고 검토할 수 있어 다른 사람에 의한 객관적 평가가 가능하다. 또한 참여자들의 경험이 다양하여 동일 문장이 아닌 간호 현장에서 전문적인 간호학 지식을 바탕으로 한 텍스트 네트워크 분석 방법은 임상증상 표현의 축적에 기여할 수 있는 연구방법으로 생각된다.

임상에서 환자의 호소 내용을 그대로 간호 기록으로 옮겨 적는 것을 보고 의학 교육의 임상 표현을 이용한 교육처럼 간호학도 임상증상 표현으로 분류하여 교육할 필요를 느껴 처음으로 도출을 처음으로 임상증상 표현 도출을 적용한 연구라는 점과, 조산 산모의 개인적 경험을 더 구조적으로 분류하기 위한 첫 시도로 간호지식 체계의 발전을 위한 전환점을 제공할 수 있다는 점에 이 연구의 의의가 있다고 생각한다.

본 연구를 통해 조산 산모들의 삶은 ‘하루하루 버티기’라는 현상으로 도출되었다. 조산 경험의 9개 구성요인은 산과적 상태, 정서적 상태, 병원 환경, 생활 스트레스, 임신 스트레스, 내과적 문제, 남편의 지지, 정보의 지지, 신체적 상태이며, 조산 산모가 경험한 최다 빈도의 키워드는 스트레스이고, 다빈도 키워드 상위 3개는 조기 진통, 개인적 특성, 조기 양막파열이다. 조산 전 임산부의 상태를 사정할 수 있는 상위 6개의 임상증상 표현은 스트레스, 배 뭉침(가진통), 태아 걱정, 양수 터짐, 진진통, 자궁 수축이다. 본 연구 결과에서 도출된 조산 관련 임상증상 표현을 산과 기록의 코딩화에 활용할 것을 제안한다. 또한 조산 경험의 9개 구성요인은 조산 관련 위험척도 개발의 틀로 연계할 수 있을 것으로 기대한다.

## Figures and Tables

**Table 1.** Characteristics of participants (N=11)

Participant’s age (yr)	Gestational age at delivery (wk/day)	PPROM	Ritodrine HCl (Yutopar)	Atosiban (Tractocile)	Birth type	Newborn		Apgar score	
						Sex	Weight (g)	1 min	5 min
37	36/6	No	Used	Not used	NSVD	Male	2830	10	10
35	35/0	Yes	Used	Used	CS	Female	2445	6	8
37	31/0	No	Used	Used	CS	Female	1700	2	8
32	36/2	Yes	Not used	Not used	CS	Female	2500	9	10
38	36/4	Yes	Used	Used	CS	Male	3040	9	10
33	36/0	No	Used	Used	CS	Female	1840	8	10
37	35/1	Yes	Not used	Not used	NSVD	Male	2500	9	10
31	32/1	Yes	Used	Used	CS	Male	1820	8	9
31	36/6	Yes	Used	Not used	NSVD	Female	2970	9	10
38	34/5	Yes	Used	Not used	NSVD	Male	2685	8	10
31	35/5	Yes	Not used	Not used	NSVD	Female	2280	9	10

CS: Cesarean section; NSVD: normal spontaneous vaginal delivery; PPROM: preterm premature rupture of membrane. Yutopar, JW Pharmaceutical, Seoul, Korea; Tractocile, Ferring, Seoul, Korea.

## Supplementary materials

Further details on supplementary materials are presented online (available at https://doi.org/10.4069/kjwhn.2020.08.08).

Supplementary data. Clinical symptom expression and contents of storytelling
