# Supplementary material for: Visualization of unstructured personal narratives of perterm birth using text network analysis
Source: Korean J Women Health Nurs. 2020 Aug 28;26(3):205–12. [Article in Korean] doi: 10.4069/kjwhn.2020.08.08 (PMC9328584; doi:10.4069/kjwhn.2020.08.08)
Supplement: Supplementary data. — Clinical symptom expression and contents of storytelling [file kjwhn-2020-08-08suppl.pdf]

Supplementary data. Clinical symptom expression and contents of storytelling

주제	하루하루 버티기
[정서적 상황]	
스트레스	'친구가 놀러 온다고 해서 신경 쓰여 과잉으로 청소를 했어요', '사기를 당했어요. 그걸로 인해 밤에 잠을 잘못 자고 이사도 해야 하고 뭐 그런 돈 같은 문제도 있으니까 이것저것 스트레스를 많이 받았지요~', '성격상 급하고 빠르고 스트레스를 많이 받는 성격이어서', '집안에서도 뭔가 정리가 딱딱 되어야 하고', '산책을 못 하니까', '아버지에게 신장이식을 해준 후 손주도 보여주고 싶어 인공수정을 했는데 한 번에 임신이 됐어요'. '친정 엄마는 공주 같아 혼자 감당했어요', '임신 중 퇴사하기 전 회사랑 트러블이 있어서', '예민하고 매사에 걱정하는 편', '의료진이 이틀에 한 번씩 부정적인 이야기를 하는데 지금은 이해하지만'
태아 걱정	'경관이 짧다 그러니까 일찍 나올까 봐 불안했죠', '단일 양막인데 탯줄이 꼬인다고 하고 아이가 살지 못한다고 하니까 청천벽력 같은', '32주에 애기 배에 복수가 차고 있다 하니까 한 주를 더 버티고 싶지만 주말에 안 좋은 상황이 일어나면 아이가 잘못될 수 있어서', '애기가 숨을 못 쉬고 죽을 수도 있는데 자가 호흡을 못 할 수도 있고 그래서 걱정을 많이 하고 맘카페 찾아보고', '하루하루 버티기가 목표였어요. 일찍 나오면 애기가 힘드니까'
[산과적 상황]	
양수 터짐	'잠잘 때 양수가 폭 터졌어요' '화장실에 가서 확인하니 락스 냄새가 나고 양도 많고', '걸을 때 주르륵 흐르고요', '걷기가 힘들 정도로 아파서 주저앉았어요. 왜 이리 아프지 몰랐는데 그게 양수가 터지려고 그랬던 것 같아요' '막 일어서려는데 근육이 막 찢어지는 느낌 있잖아요. 그때부터 물이 계속 흘러나오는 거예요', '출산하기 전부터 콧물 같은 것이 1주일 2주일 나오는 거예요. 그날도 똑같이 나오다가 새벽에 터졌어요', '양수 터질까 봐 진짜 누워만 있었어요'
배 뭉침	'주민센터에서 해주는 거 거기로 갔는데 하고 나면 무리했나? 괜히 했나? 하는 생각', '임신 기간 중 자주 뭉쳤어요', '배가 싸르르 아팠어요', '무거운 물건 들고 외근을 많이 했어요. 외근할 때 잘 뭉쳤어요'
조기 진통	'허리가 아프다', '밑이 빠질 것 같다', '축구공처럼 배가 빵빵하다', '톱으로 써는 것 같다', '전봇대로 잡아빼듯 깊숙이 아팠어요', '질 쪽에 조임 같은 느낌', '조이고 딱딱해지기만 했어요', '항문을 찌르는 느낌, 항문으로 내려가는 느낌이었어요'
자궁 수축	'미친 듯이 아팠어요', '좀 더 배꼽의 왼쪽 거기가 태반 쪽인가 움직이지 못하고 걷지도 못할 정도로 아팠어요' '너무 아파 주저앉았어요', '아침에 괜찮다가 밤에 새벽에 특히 수축이 많이 왔어요', '와이존이 빠질 것 같은 느낌이었어요', '배가 사르르 아팠어요', '엉덩이 쪽이 많이 아팠어요'
[남편 지지]	'하나부터 열까지 다 섭섭했죠', '어떻게 해달라는 게 아니잖아요. 제 말을 들어달라는 건데 모르더라구요'
[정보 지지]	'최대한 쉬라고 했는데, 집안에서 빨빨대고 돌아다녔어요', '출산 여행들 다 가잖아요', '안정하라고 했는데 이 정도 여행하는 것이면 괜찮을 줄 알았죠'. '추석이어서 연휴가 길었잖아요~ 맨날 누워있으니까 답답하니까 신랑도 있겠다 그래서 그 전날 좀 많이 돌아다녔어요'

https://doi.org/10.4069/kjwhn.2020.08.08
